# Laterally transferred elements and high pressure adaptation in *Photobacterium profundum *strains

**DOI:** 10.1186/1471-2164-6-122

**Published:** 2005-09-14

**Authors:** Stefano Campanaro, Alessandro Vezzi, Nicola Vitulo, Federico M Lauro, Michela D'Angelo, Francesca Simonato, Alessandro Cestaro, Giorgio Malacrida, Giulio Bertoloni, Giorgio Valle, Douglas H Bartlett

**Affiliations:** 1 CRIBI Biotechnology Centre and Dept. of Biology, University of Padova, Via U. Bassi 58/B, 35131 Padova, Italy; 2 Scripps Institution of Oceanography, University of California San Diego, La Jolla CA, 92093-0202, USA; 3 Department of Histology, Microbiology and Medical Biotechnology, University of Padova, Via A. Gabelli 63, 35121 Padova, Italy

## Abstract

**Background:**

Oceans cover approximately 70% of the Earth's surface with an average depth of 3800 m and a pressure of 38 MPa, thus a large part of the biosphere is occupied by high pressure environments. Piezophilic (pressure-loving) organisms are adapted to deep-sea life and grow optimally at pressures higher than 0.1 MPa. To better understand high pressure adaptation from a genomic point of view three different *Photobacterium profundum *strains were compared. Using the sequenced piezophile *P. profundum *strain SS9 as a reference, microarray technology was used to identify the genomic regions missing in two other strains: a pressure adapted strain (named DSJ4) and a pressure-sensitive strain (named 3TCK). Finally, the transcriptome of SS9 grown under different pressure (28 MPa; 45 MPa) and temperature (4°C; 16°C) conditions was analyzed taking into consideration the differentially expressed genes belonging to the flexible gene pool.

**Results:**

These studies indicated the presence of a large flexible gene pool in SS9 characterized by various horizontally acquired elements. This was verified by extensive analysis of GC content, codon usage and genomic signature of the SS9 genome. 171 open reading frames (ORFs) were found to be specifically absent or highly divergent in the piezosensitive strain, but present in the two piezophilic strains. Among these genes, six were found to also be up-regulated by high pressure.

**Conclusion:**

These data provide information on horizontal gene flow in the deep sea, provide additional details of *P. profundum *genome expression patterns and suggest genes which could perform critical functions for abyssal survival, including perhaps high pressure growth.

## Background

Piezophilic microbes have been isolated from a variety of abyssal and hadal deep-sea environments and include several psychrophilic or psychrotolerant proteobacteria, and several high temperature Euryarchaeota and Crenarchaeota [1]. While the study of these extremophiles is still in its infancy, both physiological and structural adaptations appear to be important for high-pressure life.

One moderately piezophilic, gamma-proteobacterial isolate, *Photobacterium profundum *strain SS9, has been the subject of a number of studies addressing the nature and regulation of genes important for pressure-sensing and high pressure adaptation, owing to the relative ease of its cultivation as well as its genetic tractability [1]. Here we make use of another important *P. profundum *feature, namely the availability of multiple closely related strains which differ in their pressure and temperature optima. Strain SS9 was isolated from an amphipod in the Sulu Trough at a depth of 2551 m and displays optimum growth at 28 MPa and 15°C [2]. *P. profundum *strain DSJ4 was recovered from a sediment sample obtained from the Ryukyu Trench (Japan) at a depth of 5110 m and displays its optimum growth at 10 MPa (with little change in growth at pressures up to 50 MPa) and a temperature optimum of 10°C [3]. *P. profundum *strain 3TCK was isolated from a shallow sediment sample obtained from San Diego Bay (California, U. S. A.) and exhibits optimal growth at atmospheric pressure and a broad temperature span for growth from below 0°C to above 20°C.

Recently, the complete genome sequence of strain SS9 was obtained [4]. This achievement has enabled the scaling up of the study of piezophily and, more generally, of adaptations to the deep sea (i.e., low temperature, low nutrient input, absence of sunlight), at the genomic level. In this study a microarray covering nearly the complete SS9 genome was used to investigate both the flexible gene pool (genes whose presence is variable due to insertion/deletion events) and high pressure adaptation by means of two different post-genomic approaches:

1-Using the SS9 genome as a reference, comparative genomic hybridization experiments were performed with DNA extracted from the other two *P. profundum *strains (DSJ4 and 3TCK) to identify the flexible gene pool in SS9. To determine if these genes were obtained from lateral gene transfer events or, conversely, from genomic reduction events in the other strains, their GC content, codon usage and genomic signature was analyzed.

2-Transcriptome analyses were performed as a function of pressure (0.1, 28 and 45 MPa at 16°C) and temperature (4°C vs. 16°C at 0.1 MPa). Although we have recently presented preliminary data on SS9 expression at 0.1 and 28 MPa, in this study temperature effects on gene regulation were compared with pressure effects since increasing pressure exerts some common effects with decreasing temperature in terms of membrane microviscosity and with increasing temperature in terms of protein stability [5]. Moreover the transcriptional changes identified in the 0.1 MPa vs. 28 MPa and 28 MPa vs. 45 MPa experiments were compared in order to reveal expression changes in a piezophilic bacterial species grown at supra-optimal pressure.

Finally, the results obtained from comparative genomic analyses and expression profiling experiments were combined to identify genes shared among the *P. profundum *piezophiles, absent from the piezosensitive strain, and up-regulated at high pressure. This allowed a few genes to be selected from a pool of approximately 6,000 genes whose distribution and expression characteristics suggest possible function in high pressure adaptation and thus present themselves as candidates for future genetic investigation.

## Results

### Comparison of three *P. profundum *strains

**Figure 1:**
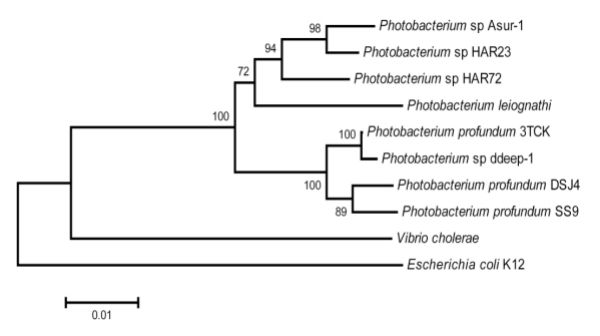
Phylogenetic tree showing the relationship of 16S rRNA gene sequence within the *Photobacterium *genus using the neighbor joining method. The scale represents the average number of nucleotide substitutions per site. Bootstrap values are from 1,000 replicates and shown for frequencies above the threshold of 50%. The phylogenetic tree was created using *E. coli *and *V. cholerae *as outgroups.

Amplification and analysis of the 16S rDNA from strain 3TCK revealed that it is 97.7% identical at the 16S level with *Photobacterium profundum *SS9 and 98.7% identical to *P. profundum *DSJ4, suggesting that they are all members of the same species. Figure 1 shows a 16S rRNA-based phylogenetic tree demonstrating the relationship among selected *Photobacterium *species, including the three *P. profundum *strains selected for this study. Growth curves showed that 3TCK is psychrotolerant and piezo-sensitive. However it grows at higher temperatures than *P. profundum *SS9 and DSJ4 (data not shown) and has faster growth rates at 0.1 MPa.

### Genomic comparison between different *P. profundum *strains

The first question that arises from the genomic comparison between SS9 and the other two strains is: "how many SS9 genes are missing or highly divergent in the 3TCK and DSJ4 genomes?". 544 ORFs were determined to be absent in 3TCK strain genome, 313 (9.1% of the ORFs located on chr1) belong to the SS9 chr1 and 231 (11.5% of the ORFs located on chr2) to chr2. 562 ORFs are absent in the DSJ4 genome, 292 (8.5%) are located on SS9 chr1 and 270 (13.5%) on chr2.

**Figure 2:**
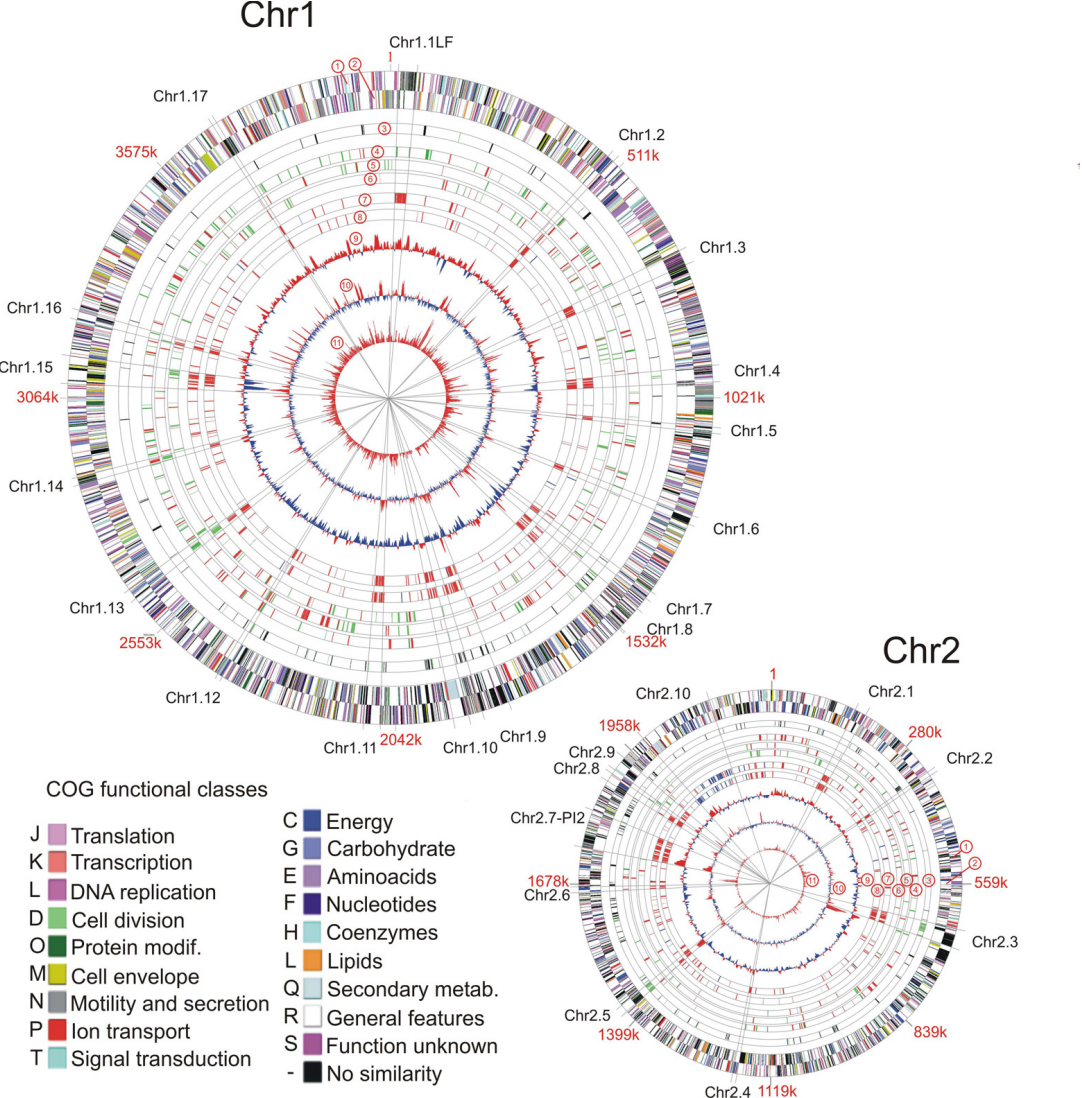
Genomic organization of the three *P. profundum *strains compared with expression level and differentially expressed genes obtained from microarray experiments. Form the outside inward circles represent: 1, 2) predicted protein-coding ORFs on the plus and minus strands of SS9 genome (colours were assigned according to the colour code of the COG functional classes); 3) transposon-related proteins (black bars) and genes showing similarity with phage proteins (green bars); differentially expressed genes in 4) 0.1 MPa vs. 28 MPa microarray experiment (green and red bars represent genes up-regulated respectively at 0.1 MPa and 28 MPa); 5) 45 MPa vs. 28 MPa (green and red bars represent genes up-regulated respectively at 28 MPa and 45 MPa); 6) 4°C vs. 16°C (green and red bars represent genes up-regulated respectively at 16°C and 4°C); 7) genomic regions absent (red bars) or duplicate (blue bars) in 3 TCK strain (compared to SS9 strain) and 8) in DSJ4 strain (compared to SS9); 9) GC content variation; 10) 10 kbp windows genomic signature compared to total genomic signature; 11) absolute expression level at 28 MPa obtained from microarray experiments.

An interesting aspect of these data is that for both strains the percentage of missing/divergent regions is higher on chr2 than chr1. This indicates that chr2 (Figure 2) contains a proportionally larger flexible gene pool and that it has been the target of more gene transfer events for its size (~2.2 Mbp) than chr1 (~4.1 Mbp) that contains the most "established" genes. This is also true for other *Vibrionaceae *genomes [6].

**Figure 3:**
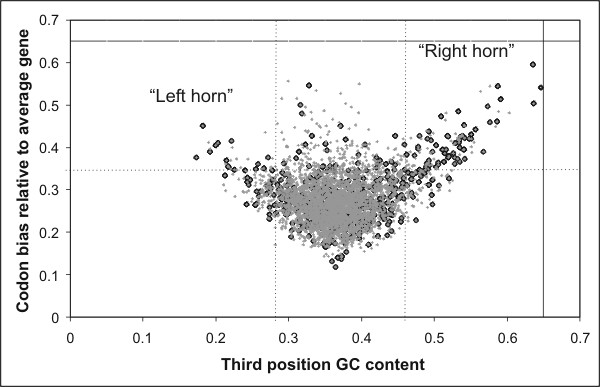
Evidence for lateral gene transfer in SS9. Each *P. profundum *gene of ≥ 200 codons is represented in this graph by a point with co-ordinates corresponding to its codon bias relative to average gene and G+C frequency of codon site three. Genes having G+C frequency lower than 0.28 and codon bias higher than 0.35 correspond to the left horn. Genes having G+C frequency higher than 0.46 and codon bias higher than 0.35 correspond to the right horn. Genes absent in 3TCK and/or DSJ4 genomes (highlighted in black) are more frequent in horn regions (64% in left horn, 66% in right horn) respect to the middle region (10% of ORFs). The low number of strains analyzed leads to an underestimation of the laterally transferred regions in SS9 and this probably accounts for those genes localized in left and right horn which are present in all three *P. profundum *strains analyzed.

In order to define if the regions absent in the 3TCK-DSJ4 strains could be considered to have been acquired by horizontal gene transfer we performed three different analyses: GC content variation (Figure 2, 9^th ^circle), tetranucleotide composition (genomic signature) (Figure 2, 10^th ^circle) [7,8] and codon bias relative to the average gene versus S3 percentage (G+C content of codon site 3) (Figure 3 and Additional file 1) [9].

Taken together these three different analyses were able to identify a large number of potentially laterally transferred regions. For example, a region named Chr1.11 (named for its chromosome location and clockwise order of position, Figure 2) has an altered tetranucleotide composition but its GC content is similar to the surrounding regions and it has a "normal" codon bias relative to the average gene versus S3 percentage (data not shown). Conversely, region Chr2.1 is only characterized by a slight GC content variation. The results obtained from these analyses are discussed in-depth below.

A BLASTP similarity search of SS9 proteins identified various phage-related proteins, mostly encoded in three regions named Chr1.8, Chr2.3 and Chr2.5. Microarray data obtained by comparing the SS9, 3TCK and DSJ4 genomes (Figure 2) confirmed that these genomic portions are absent in both the 3TCK and DSJ4. These regions present characteristics typical of a genomic island (GI): (1) GC content anomalies, (2) altered codon bias, (3) insertion at the 3'-end of a tRNA gene (tRNA-N) and (4) presence of a gene encoding an integrase at one end (Table 1) [10].

**Table 1:** General characteristics of putative horizontally transferred elements found in the genome of *P. profundum *SS9 strain.

**Element**	**Absent in strain**	**Start-end position**	**Size (kbp)**	**GC content percent (chr1_42%; chr2_41.2%)**	**tRNA**	**Integrase-like genes at the end**	**Expression level**	**Relevant resident genes**
Plasmid	3TCK DSJ4	1–80,033	80	44%	NO	NO	moderate (4,458)	
Chr1.1-LF	3TCK	13,500–51,360	38	45%	NO	NO	low (1,079)	LF-lateral flagella coding region; there are two genes up-regulated at 0.1 MPa at both ends (PBPRA0013- PBPRA0050).
Chr1.2	3TCK DSJ4	479,200–490,850	11.6	44%	NO	NO	low (2,623)	Xylose transport and metabolism; one gene up-regulated at 4°C (PBPRA0466).
Chr1.3	3TCK	717,590–751,055	33.4	41%	NO	NO	moderate (3,879)	
Chr1.4	3TCK DSJ4	957,470–983,750	26.3	40%	YES	YES (PBPRA0877)	moderate (3,744)	
Chr1.5	3TCK DSJ4	1,081,250–1,093,850	12.6	41%	NO	NO	low (1,094)	
Chr1.6	DSJ4	1,262,100–1,281,040	21.7	41%	NO	NO	low (1,111)	One up-regulated gene at 0.1 MPa near 5'-end (PBPRA1136).
Chr1.7	DSJ4	1,436,830–1,453,450	16.6	42%	NO	NO	low (2,432)	ORFs involved in amino acid metabolism and transport.
Chr1.8	3TCK DSJ4	1,482,130–1,528,980	46.8	40%	NO	YES (PBPRA1336)	moderate (3,453)	12 phage-related proteins and genes involved in tryptophan transport and metabolism.
Chr1.9	3TCK DSJ4 (smaller)	1,803,270–1,847,340; 1,803,270–1,837,500	44.1 3TCK; 34.2 DSJ4	40% 41%	NO	NO	moderate (4,586)	One gene up-regulated at 28 MPa (vs. 0.1 MPa) (PBPRA1598) and numerous transposases.
Chr1.10	3TCK-DSJ4	1,887,600–1,921,860	34.2	42.9%	YES	NO	low (2,509)	Genes involved in fatty acid synthesis and tryptophan biosynthesis.
Chr1.11	3TCK-DSJ4	2,063,315–2,097,000	33.7	40%	YES	NO	moderate (3,276)	One gene (PBPRA1810) up-regulated at 16°C.
Chr1.12	DSJ4	2,399,560–2,410,040	10.5 DSJ4	40%	YES	NO	moderate (4,179)	Three genes differentially expressed (PBPRA2084, PBPRA2086, PBPRA2087).
Chr1.13	3TCK (smaller) DSJ4	2,640,200–2,650,840	10.6 3TCK 17.7 DSJ4;	41%	YES	NO	moderate (12,480-7,436)	Gene cluster involved in tricarboxylic acid fermentation and transport; some genes were up-regulated at 16°C and/or 0.1 MPa.
Chr1.14	DSJ4	2,892,510–2,901,270	8.8	45.9%	NO	NO	high (11,847)	Genes involved in pilus assembly, three genes up-regulated at 28 MPa (vs. 0.1 MPa) and/or down-regulated at 45 MPa (PBPRA2498, PBPRA2499, PBPRA2505).
Chr1.15	3TCK-DSJ4	3,104,770–3,110,020; 3,145,100-3,145,095	5.2+ 29.3	29%–37%	YES	NO	moderate (9,965)	flm genes; two genes down-regulated at 4°C (PBPRA2692, PBPRA2701).
Chr1.16	3TCK-DSJ4	3,220,530–3,230,610	10	43.9%	NO	NO	moderate (4,394)	Genes coding for a phosphotransferase system cellobiose-specific; one gene up-regulated at 4°C (PBPRA2779).
Chr1.17	3TCK-DSJ4	3,706,515–3,712,920	6.4	45.3%	NO	NO	moderate (6,448)	
Chr2.1	3TCK-DSJ4	159,600–184,460	24.9	43.4%	NO	NO	low (2,659)	Genes involved in pentose and glucuronate interconversions and PTS system.
Chr2.2	3TCK-DSJ4	342,190–349,500	7.3	43.2%	NO	NO	low (1,741)	Very small region, no orthologous in other Vibrio species
Chr2.3	3TCK-DSJ4	637,160–679,550	42.4	41.1%	NO	YES (PBPRB0551)	low (2,485)	12 phage-related proteins; similarities with Gifsy -1, Gifsy -2 prophages protein.
Chr2.4	DSJ4	1,179,200–1,191,215	12	42.8%	NO	NO	low (1,191)	Phosphotransferase system (PTS) N-acetylgalactosamine-specific genes.
Chr2.5	3TCK (smaller) DSJ4	1,419,870–1,427,600; 1,419,870–1,439,670	19.8	46.2%	NO	YES (PBPRB1271)	low (1,737)	12 phage-related proteins.
Chr2.6	3TCK-DSJ4	1,661,040–1,685,300	24.3	39.2%	YES	NO	moderate (3,201)	Four transposases in the central part; genes involved in pentose and glucuronate interconversions; genes involved in C4-dicarboxylate transport system
Chr2.7-PI	3TCK DSJ4	1,745,750–1,814,900	69.1	44.8%	NO	YES (PBPRB1675)	low (2,060)	This region could be derived from a plasmid integration.
Chr2.8	3TCK-DSJ4	1,872,240–1,891,940	19.7	40.4%	NO	NO	moderate (4,347)	Genes involved in C4-dicarboxylate transport system and in pentose and glucuronate interconversion pathway.
Chr2.9	DSJ4	1,901,330–1,928,060	26.7	41.9%	YES?	NO	moderate (4,074)	Highly discontinuous region.
Chr2.10	3TCK-DSJ4	2,013,000–2,087,900; 1,975,200–2,095,800	~108 3TCK; ~145 DSJ4	41.2%–42.7%				This region is probably duplicated in the test strains (DSJ4 and 3TCK.

Low, moderate and high expression values are calculated on the basis of the microarray clones fluorescence intensity and correspond respectively to values lower than 3,000, comprise between 3,000 and 10,000 and higher than 10,000 arbitrary fluorescence units. Negative controls clones have a mean fluorescence value of 377 arbitrary fluorescence units. As an example, clones localized in the ATP synthase operon have a mean fluorescence value of 36,100 arbitrary fluorescence units and clones localized in polar flagellum cluster have a mean fluorescence value of 10,200. For region Chr2.10 mean fluorescence value are not reported because it is very large and a mean fluorescence value has little significance.

Chr1.8 (46.8 kbp) has an altered tetranucleotide composition and presents twelve phage-related proteins, one of these (PBPRA1336) encodes a putative integrase protein. Nevertheless this region lacks the other two characteristics of a typical GI: GC content anomalies and the presence of a tRNA gene at one end. This region also contains two genes, encoding tryptophanase (TnaA) (PBPRA1344) and a hypothetical tryptophan-specific transport protein (PBPRA1345), involved in tryptophan transport and metabolism, and 27 hypothetical or conserved hypothetical proteins.

The region Chr2.3 is located on chr2, spans approximately 42.4 kbp, has no GC content anomalies (41.1%) but has a slightly altered tetranucleotide composition. Chr2.3 contains twelve ORFs that have similarity with phage proteins, one of these being a hypothetical integrase (PBPRB0551). Moreover it encodes a putative NAD(P)H oxidoreductase (PBPRB0548) and a putative TrkA family protein (glutathione-regulated potassium efflux system protein) (PBPRB0550). Furthermore PBPRB0559 gene has similarities with enterohemolysin 1, a gene also present in the Gifsy-1 prophage, and PBPRB0560 has similarities with exodeoxyribonuclease of the Gifsy-2 prophage of *Salmonella typhimurium *LT2 [11].

Chr2.5 region appears to be completely absent in DSJ4 whereas only the first part is absent in 3TCK. Chr2.5 contains a hypothetical integrase gene (PBPRB1271), twelve phage related proteins and various hypothetical proteins. The higher GC content (46.2%) of Chr2.5 suggests that it has been acquired more recently than Chr1.8 and Chr2.3.

A large part of the genes located in the Chr1.8, Chr2.3 and Chr2.5 regions clearly lacks orthologous genes in others bacteria [12] such as *V. cholerae*, *V. vulnificus *(strains CMCP6 and YJ016), *V. parahaemolyticus*, *V. fisheri*, *E. coli*, and *B. subtilis*. The high number of hypothetical proteins encoded in these regions suggests that these loci could have been acquired from bacteria still unknown. Consistent with its recent acquisition Chr2.5 presents an altered codon bias and a large percentage of its ORFs are located on the right horn of a graph of the codon bias versus the GC content frequency in third position (Additional file 1).

A large 69.1 kbp element (Chr2.7-PI for Plasmid Integration) is present only in strain SS9 and seems to be the result of plasmid integration into the chromosome. This region has a high GC content and altered codon bias and genomic signature. Various bacterial conjugation factors (TrbCDEJBFGI and TraFLGIKL) are present in this element. These genes are typically found in widespread conjugationally transmitted plasmids [13]. This element carries a large number of genes, but of particular interest is a multidrug efflux system (PBPRB1635, PBPRB1637, PBPRB1638).

SS9 also contains a 80 kbp plasmid (named "plasmid" in Table 1) that shows similarity with *V. vulnificus *plasmid YJ016 at least in the region spanning the genes related to conjugation. This plasmid is absent in both DSJ4 and 3TCK and presents characteristics of horizontally transferred DNA, in fact various ORFs belonging to this element are localized to the right horn of Figure 3 (see also Additional file 1).

PCR examination for the presence of this plasmid in various laboratory derivatives of *P. profundum *SS9 has revealed that it can be lost: strain TW30 [14] is a *toxR*^- ^derivative of DB110 [15] which lacks the plasmid (Additional file 2), yet TW30 exhibits no pressure or temperature growth defects. This raises the question of the function of the genes located on the plasmid which must be playing some role in environmental adaptation for the plasmid to be retained [16].

One of the most interesting results obtained from the annotation process was the finding that in the SS9 genome there are two flagellar clusters. One of these (tentatively identified as the polar flagella cluster, PF) is located on chr1 between 993676 and 1046789 bp and the second cluster (tentatively identified as the lateral flagella cluster, Chr1.1-LF) is localized on chr1 near the origin between 13496 and 49254 bp. Similarity searches revealed that the PF region contains most of the genes involved in polar flagellum assembly, with a gene organization typical of a *Vibrionaceae *polar flagellar cluster [17]. Chr1.1-LF contains all the genes involved in lateral flagellar synthesis and most of the genes localized in this region have similarity with the flagellar cluster of *V. parahaemolyticus *that codes for lateral flagella [18]. This region is absent only in 3TCK strain (Figure 2).

Finally, the data on variable regions was compared with the transcriptome results to discern if any of the horizontally acquired genes might be indicated to perform a role in pressure or temperature adaptation. The absolute fluorescence value from microarray analysis indicates that the expression levels of most of these genes was quite low. Indeed, only three regions in chr1 (Chr1.13, Chr1.14, Chr1.15) and two in chr2 (Chr2.8, Chr2.9) had fluorescence values higher than the mean fluorescence level of the entire chromosome (Table 1 and Figure 2, 11^th ^circle).

Region Chr1.12 is absent in DSJ4 (and in 3TCK this region is smaller) and has three genes, PBPRA2087 (hypothetical protein), PBPRA2084 (putatively evolved beta-D-galactosidase, alpha subunit) and PBPRA2086 (putative oxidoreductase), differentially expressed in pressure experiments.

Region Chr1.13 is absent in both strains and contains a large gene cluster involved in the tricarboxylic acid fermentation and in the cleavage of citrate to oxaloacetate and acetate. This region contains six genes up-regulated at 16°C and/or 0.1 MPa (PBPRA2289, PBPRA2292, PBPRA2295, PBPRA2298, PBPRA2300, PBPRA2303) (Additional file 3).

Region Chr1.14 is absent in DSJ4 and contains genes involved in pilus assembly, some of these are up-regulated at 28 MPa (vs. 0.1 MPa) and down-regulated at 45 MPa (PBPRA2498, PBPRA2499, PBPRA2505).

Finally, region Chr1.15 is lacking in both of the comparison strains and contains genes having a high expression level at 28 MPa, some of which are differentially expressed at 28 MPa and 4°C (PBPRA2692, PBPRA2701, PBPRA2710). This region contains *flm *genes that in other bacteria are involved in LPS O-Ag biosynthesis and flagellar filament assembly [19]. Interestingly changes in LPS O-antigen structure have been observed in *Yersinia pestis *KM218 grown at low temperatures [20]. This element has very low GC content (29%–37%), altered genomic signature and most part of its genes are localized to the left horn of the graph reported in supporting online material (Table 1, Figure 3 and Additional file 1), thus supporting the idea that it could have been laterally acquired.

It is curious that some of the variable regions differentially expressed in pressure experiments are lacking or are highly divergent in both 3TCK and DSJ4. This indicates that although genes located in these regions could be involved in the high pressure response of SS9, they are not essential to it and other *P. profundum *strains will achieve piezophily with different strategies. Moreover, genes differentially expressed at 28 MPa or 45 MPa, but present in both DSJ4 and 3TCK, could be beneficial but not sufficient for high pressure adaptation.

Considering only pressure regulated genes belonging to the group absent in 3TCK and/or DSJ4, 29 genes are absent in both strains but only 9 genes are absent in 3TCK strain alone (Additional file 3 and Additional file 4). These data were obtained using a profile search with JExpress software [21]. Of these 9 genes, 6 are up-regulated at 28 MPa (vs. 0.1 MPa) and/or 45 MPa (PBPRB0026 hypothetical sensor protein TorS; PBPRA0776 hypothetical protein; PBPRA1912 hypothetical protein; PBPRA2251 hypothetical ABC transporter, periplasmic solute-binding protein, family 5; PBPRA2252 hypothetical ABC transporter, permease protein; PBPRA2573 putative long-chain fatty acid transport protein).

In the SS9 genome we found two genes for TorS proteins (PBPRA1232 and PBPRB0026). Only one of these (named PBPRB0026) is differentially expressed at 28 MPa and this gene is also absent in 3TCK strain. TorS is able to regulate various genes in response to trimethylamine N-oxide (TMAO) [22], in particular it regulates TMAO reductase (PBPRA1467) that is also up-regulated at 28 MPa. It is conceivable that trimethylamine reduction increases the pH of the cytoplasm and, for this reason, other genes identified with microarray experiments, such as tryptophanase (PBPRB0382 and PBPRA2532) increase their expression in order to counter this alcalinization. Alternatively, since no TMAO was added to the SS9 cultures used for the microarray experiments, the second TorS could also be responding to an as yet undiscovered signal.

In addition to these six genes, others could perform an important role in high pressure adaptation. We expect that a high number of genes and proteins are regulated at the post-transcriptional level and have an important role in high pressure adaptation but these studies are beyond the scope of this paper. Moreover protein structural adaptations, that were not considered in this analysis, could also have great importance for SS9 piezophily.

### Transcriptome analysis of *P. profundum *strain SS9 under different pressure and temperature conditions

In a previous paper a preliminary analysis was presented for genes differentially expressed at 28 MPa (the optimum growth pressure for SS9) versus 0.1 MPa, highlighting a stress response at low pressure, an heavy involvement of membrane transporters in pressure adaptation and an increased expression at 28 MPa of genes involved in the Stickland reaction and TMAO reduction. Nevertheless, additional transcriptional responses remain to be elucidated including the response to low temperatures and the effect of supraoptimal pressures (45 MPa) on SS9. To better elucidate these points expression profiling was performed at different temperatures (4°C vs. 16°C) and different pressures (28 MPa vs. 45 MPa). To clarify the role of specific biological processes on temperature and pressure adaptation, we also performed a Gene Ontology analysis [23] on differentially expressed genes using specific software such as GoMiner [24] and FatiGO [25].

### Analysis of co-regulated genes in pressure and temperature experiments

The rationale for temperature experiments was the comprehension of which genes are co-regulated during pressure and temperature changes. It is known that low temperature and high pressure have similar effects on some biological structures (for example membranes) [5]. We found 36 (43 considering also those that are co-regulated between 45 MPa and temperature) out of 319 genes that share a similar expression pattern between temperature (16°C vs. 4°C) and pressure (0.1 MPa vs. 28 MPa) experiments, a number higher that expected only by chance. In fact, the number of up-regulated genes at 28 MPa and at 4°C is respectively 101 and 75 over 4752 (the total number of genes covered by our array). If the two conditions are independent, the expected number of genes that are up-regulated both at high pressure and low temperature could be estimated as (101/4752)*(75/4752)*4752 = 1.6. Similarly we could estimate the number of down-regulated genes at 28 MPa and 4°C as (108/4752)*(85/4752)*4752 = 1.9. So the total number of genes expected to be up- or down-regulated in both the conditions considered (3.5) is approximately 1/10th of the differentially expressed genes observed. Therefore microarray experiments corroborate the hypothesis that high pressure and low temperature have a overlapping effects on gene expression.

The SS9 genome sequence reveals the presence of two iron transporters: one (PBPRB0373-PBPRB0375) that is up-regulated at 28 MPa (vs. 0.1 MPa) and at 4°C, while the other one (PBPRA3182, PBPRA3183, PBPRA3184) is up-regulated at 0.1 MPa (vs. 0.1 MPa) and at 16°C. Iron accumulation in organisms that live in the ocean environment is difficult [26] and the evolution of two alternative transporters could be important in order to survive under different physical conditions.

The presence of different isoforms of the same transporter that work at different pressure and temperature conditions is not limited to iron transporters. ORFs PBPRA0098-PBPRA0101 code for a hypothetical oligopeptide transporter and are up-regulated at 0.1 MPa, while ORFs PBPRA2251-PBPRA2254 code for a different oligopeptide transporter that is up-regulated at 28 MPa (vs. 0.1 MPa) and 45 MPa (vs. 28 MPa). Other oligopeptide transporters seem to have the same behaviour such as those codified by PBPRA0521-PBPRA0525 and PBPRA2934-PBPR2938, the first being up-regulated at 28 MPa (vs. 0.1 MPa) and 4°C, while the second is weakly up-regulated at 0.1 MPa.

There is an entire region (Table 1; region Chr1.13) containing a large number of genes that are up-regulated both at 0.1 MPa and at 16°C. This region was also identified in the above genome comparisons because it is absent in both 3TCK and DSJ4 strains.

### Analysis of co-regulated genes in 28 MPa and 45 MPa experiments

The expression profile of SS9 at 28 MPa (the optimal growth pressure) was also compared with that at 45 MPa.

Interestingly the 45 MPa vs. 28 MPa expression profile comparison revealed only 68 differentially expressed genes (33 up-regulated and 35 down-regulated), in contrast to the high number of differentially expressed genes between 28 MPa and 0.1 MPa (101 up-regulated and 108 down-regulated). Of these 68 differentially expressed genes, only 31 were specific for very high pressure adaptation, the remaining 37 also being expressed under other environmental conditions tested. This result indicates that SS9 undergoes a heavy reorganization in gene expression between atmospheric pressure and 28 MPa, while this is not seen moving from 28 MPa to 45 MPa.

A Gene Ontology search with GoMiner software [24] indicated that among the 31 genes specific for very high pressure adaptation there is an enrichment of genes involved in arginine metabolism (GO: 0006525), catabolism (GO: 0006527) (Additional file 5) and transport (PBPRA2073-PBPRA2076). Experiments at 45 MPa were also useful in identifying genes whose expression follows the direction of pressure variation, being up-regulated or down-regulated both at 28 MPa (compared to 0.1 MPa) and at 45 MPa (compared to 28 MPa). Twenty one genes matched this expression profile (Additional file 3).

One of these genes encodes a putative delta-9 fatty acid desaturase (PBPRB0742). High pressure increases the rigidity of membranes [27,28], and for this reason genes such as the putative delta-9 fatty acid desaturase presumably were up-regulated in order to increase the membrane unsaturation and thus membrane fluidity. Despite the fact that much is known about membrane modification in response to pressure variation in SS9 [29,30], these experiments reveal the possible involvement of a previously unrecognized gene in fatty acid unsaturation. This is particularly noteworthy because fatty acid unsaturation is critical to high pressure growth of SS9.

A search performed using GoMiner software [24] on differentially expressed genes obtained in the 28 MPa vs. 0.1 MPa experiments and in the 28 MPa vs. 45 MPa experiments indicated that transport is one of the main biological processes involved (Additional file 5). Similar result was obtained using FatiGO software [25] (data not shown).

Moreover six of the ORFs that are up-regulated both at 28 MPa (vs. 0.1 MPa) and 45 MPa (vs. 28 MPa) are involved in transport processes (GO:0006810) (PBPRB1789, PBPRB1788, PBPRA2251, PBPRA1366, PBPRA0555, PBPRA1297). As described in the previously published 28 MPa versus 0.1 MPa experiments [4], transport is strongly influenced by pressure, probably due to the effect of pressure on membrane modification and because of the pressure influence on the activation volume ΔV^# ^(the difference between the transition state volume and the initial volume in the system at equilibrium) of the transport process.

## Discussion

In the microbial world genetic material can be transferred between species by several mechanisms involving conjugative plasmids, phages, phage-like elements or transposable elements [31]. These elements allow exchange among bacteria of a flexible gene pool encoding additional functions that usually are not essential for bacterial growth, but which provide advantages under particular conditions. Despite the strong pressure on bacteria to maintain a small genome size by deleting the more expendable sequences from their genomes, advantages provided by some regions allow the maintenance of a flexible gene pool [32,33]. Recently [34] it was demonstrated that in the case of *Xylella fastidiosa *a large part of its flexible gene pool seems to be important in order to explain the broad host range of this phytopatogen. This despite the lack of expression of these genes under the culture conditions examined. Using clones selected from the SS9 genome sequencing project and from a specific genomic library with short inserts, a microarray covering large part of the SS9 genomic sequence (78.0% on chr1, 69.7% on chr2 and 79.3% on plasmid) was prepared and used to identify variable regions in three *P. profundum *strains. The lower coverage of chr2 was due to the higher frequency of repeated regions present on this chromosome, identified using Phred/Phrap software during finishing step. Clones were selected which lacked these regions in order to avoid cross hybridization.

This type of analysis has previously been applied to other bacterial species [34-37] but this is the first report regarding a microbial species containing members adapted to a high pressure environment. In fact, using genomic DNA derived from deep-sea (SS9 and DSJ4) and coastal (3TCK) isolates we were able to identify 28 regions that are absent or highly divergent in DSJ4 and 3TCK strains plus a large number of small regions scattered over the entire SS9 genome.

*In silico *analysis performed on the SS9 genome sequence reveals that some of these regions (for example Chr1.15, Chr2.5, Chr2.7-PI) show differences in GC content, codon usage and genomic signature (see also Table 1). There are also regions, such as Chr1 1634244–1651614 bp (containing the omega-3 polyunsaturated fatty acid synthase, *pfa*, operon), that appear as alien DNA from these analyses, but which are present in all three strains analyzed. While *pfa *mutants are not pressure-sensitive in laboratory culture [29], it is likely that the *pfa *operon is involved in high pressure adaptation under some deep-sea conditions because omega-3 polyunsaturated fatty acids (PUFAs) do increase in abundance in SS9 membranes with increasing pressure [29,30] and because such fatty acids are known to modify membrane fluidity in response to hydrostatic pressure and temperature.

These results indicate that bioinformatics and genomic microarray analysis can be merged in order to obtain a more comprehensive picture of the flexible gene pool in bacteria. Moreover, some of the variable regions identified by microarray analysis but not by bioinformatic analysis could derive from genomic reduction and not from lateral gene transfer.

On the other hand, when the two separate analyses are in accordance with each other, lateral gene transfer is the most plausible explanation. This is the case for the 80 kbp plasmid. However, the adaptive value of this plasmid to SS9 remains a mystery. It contains no obvious essential genes and can be lost during laboratory cultivation without a detectable phenotypic change.

A region that is particularly interesting is Chr1.1-LF, consisting mainly of a gene cluster present in SS9 and DSJ4 strains and coding for a second flagellar motility system. It is known that bacterial motility in the sea is a commonly expressed phenotype [38]. This character is important in enhancing bacteria-organic-matter coupling. Moreover, high speed swimming in bacteria may reduce the ability of protozoa to graze on them [39]. Some bacteria, such as *Vibrio parahaemolyticus*, posses dual flagellar systems that operate under different circumstances: a polar flagellum allows motility in liquid environments (i.e. swimming), while multiple lateral flagella allow translocation over surfaces or in viscous media [40,18]. The role of the second motility system in SS9 and DSJ4 motility is currently being investigated.

The three genomic islands Chr1.8, Chr2.3 and Chr2.5 are likely to be prophages in the *P. profundum *SS9 genome. It has been suggested that prophages might carry genes beneficial for survival of the host in a selective environment [41]. In this respect the presence, in these regions, of metabolic genes such as NAD(P)H oxidoreductase (PBPRB0548), a putative TrkA family protein (glutathione-regulated potassium efflux system protein) (PBPRB0550) and tryptophanase (PBPRA1344) might be meaningful. The latter gene has two other paralogues in the SS9 genome one of which (PBPRA2532) is pressure regulated suggesting some role in deep-sea adaptation.

Recently [4] we have shown that when moving from 28 MPa to 0.1 MPa, SS9 undergoes various modifications in metabolic processes. At 28 MPa up-regulation of genes involved in the Stickland reaction (an amino acid fermentation process typical of anaerobic bacteria such as *Clostridiales *and *Spirochetales*) and of TMAO reduction occurs.

Another interesting metabolic modification involves genes of the citrate fermentation pathway located on region Chr1.13. In *Leuconostoc paramesenteroides *the citMCDEFGRP operon, involved in citrate utilization, is located on a plasmid [42]. In SS9 these genes are down-regulated both at 28 MPa and at 4°C, moreover this region is absent both in DSJ4 and 3TCK genomes. The reason for the altered expression of this pathway in SS9 is not clear but pressure could favour some metabolic processes only on the basis of chemical-physical parameters. Some of these genes are absent in 3TCK and DSJ4 and this could reduce the growth rate of these strains at high pressure. In fact reactions accompanied by large volume variation are greatly influenced by pressure, but even if the value of ΔV (the difference between the final and initial volume in entire system at equilibrium, reaction volume) or ΔV^# ^(apparent volume change of activation or activation volume) is known, it is still difficult to predict how elevated pressure will affect metabolic pathways in living organisms [43].

As previously reported [4], low pressure induces various stress responses reflected by the up-regulation of chaperones (PBPRA0698, PBPRA1023, PBPRA1484, PBPRA3387) and DNA repair enzymes (PBPRA3513, PBPRB0986, PBPRA0694). This stress response is not present at 45 MPa despite being supraoptimal for SS9 growth. Perhaps low pressure affects protein folding because these SS9 proteins are adapted to high pressure and this effect is not evident in high pressure experiments (at least up to 45 MPa). Another curious result stemming from the 45 MPa transcriptome experiment is the apparent reduction in arginine biosynthesis and transport at high pressure relative to that at 28 MPa. It could be that this large amino acid is selected against within much of the protein pool present at this high pressure, in contrast to the thermophilic and piezophilic Archaea *Pyrococcus abyssi *which has a higher arginine content in its proteins respect to the non-piezophilic bacterium *Pyrococcus furiosus *[44]. Alternatively, other genes could govern arginine utilization at high pressure.

Transport seems to be the cellular process most influenced by pressure, at least considering the number of regulated genes belonging to this category. This influence could be due to volume changes associated with the transport process [45]. Also temperature variation influences the transport process, which could be due to alterations of transporters efficiency induced by membrane fluidity modifications. It is interesting to note that some transporters are present in two or more copies in the SS9 genome and therefore could work at specific pressures and temperatures.

Likewise, SS9 may choose among different metabolic processes (amino acid reduction, TMAO reduction, citrate fermentation pathway, etc.) as a function of pressure in order to optimize energy gain. Other mechanisms previously described [4], such as the influence of pressure on enzymes involved in complex polysaccharides utilization (PBPRA0480, PBPRA2198, PBPRB1016), support this hypothesis. The ability of SS9 to choose between different transporters or metabolic strategies could also be related to the fact that SS9 is not an obligate and narrow spectrum piezophile, but is able to grow over a large range of pressures.

## Conclusion

Our findings on genome organization and transcriptional activity of different *P. profundum *strains depict a high level of genetic diversity, where variable regions influence extremely important processes such as motility and energy production. But, due to the complexity of deep-sea environment, characterized by a peculiar combination of chemical-physical parameters and nutrient resources, it is difficult to assign a role to all the variable regions present only in the pressure adapted strains.

Expression studies on *P. profundum *SS9 performed at different pressure and temperature conditions reveal a complex adaptation network involving a great number of membrane transporters, metabolic processes, and amino acid biosynthesis and membrane modification enzymes. Some of these genes are located on variable regions.

The pressure-regulated genes unique to piezophilic *P. profundum *strains are likely to be fruitful targets of future genetic investigations into genes which facilitate growth and survival under deep-sea conditions.

## Methods

### Origin of the isolates and growth conditions

*Photobacterium profundum *SS9 and *Photobacterium profundum *DSJ4 have been previously described [2,3]. *Photobacterium profundum *strain 3TCK was isolated by the University of California San Diego Environmental Microbiology Laboratory class during a cruise on board the RV Robert Gordon Sproul on April 17, 1999. It was obtained from a superficial sediment sample within San Diego Bay following dilution plating onto a modified K Medium [46] and incubation at 23°C for several weeks. One of the resulting colonies subsequently analyzed by 16S rRNA sequence analysis was determined to be a new strain of *P. profundum*. The 3TCK 16S rDNA was amplified and sequenced using general eubacterial primers [47] on a MegaBACE 1000 automated sequencer (Amersham Biosciences, Piscataway, NJ) according to manufacturer's instructions.

Maintaining bacterial cultures in balanced growth at high pressure, when the cultures are separated from the investigator by ~2 cm of stainless steel, is extremely challenging. Because of these constraints it was not possible for us to serially culture cells under anaerobic, high pressure conditions (at least not without regularly shocking cultures with decompression for several minutes), and thus only one round of culturing was used. Overnight log phase atmospheric pressure cultures of *P. profundum *SS9 cells were grown in Difco Marine 2216 Broth supplemented with 20 mM glucose and 100 mM HEPES, pH 7.5. These cells were diluted 250-fold into the same medium, transferred to polyethylene bulbs, sealed with no air space and placed inside pressure vessels. Cultures were then rocked gently back and forth in a large refrigerated water bath shaker at either atmospheric pressure, at 28 MPa or at 45 MPa. Pressure was applied using a hand operated Haskel pump and a quick fit connector to the pressure vessel. With this system pressurization requires approximately 1 min and decompression ~1 second.

Under these culture conditions the cells quickly used up most of the available dissolved oxygen, as reflected by resazurin dye reduction analyses. After about 20–30 hours the cultures were harvested. In all cases cells were obtained from early log phase cultures (optical density at A600 nm of 0.1–0.21). The cells were immediately pelleted and RNA was immediately extracted and stored in 75% ethanol at -80°C.

For genomic experiments approximately 1 liter of bacterial cells was harvested by centrifugation for 15 min at 5,000 × g and the pellet was resuspended in 5 ml buffer A (50 mM Tris, 50 mM EDTA, pH 8.0). The suspension was incubated overnight at -20°C. Then 500 μl of buffer B (250 mM Tris, pH 8.0, 10 mg/ml lysozime) were added to the frozen suspension, which was thawed at room temperature and subsequently incubated on ice for 45 min. At this point 1 ml of buffer C (0.5% SDS, 50 mM Tris, 400 mM EDTA, pH 7.5, 1 mg/ml Proteinase K) was added and the suspension was incubated at 50°C for 60 min. Additional 750 μl of buffer C were added followed by another 30 min incubation at 50°C. The genomic DNA was extracted twice with 5 ml. of phenol:chloroform:isoamyl alcohol (24:24:1), and precipitated with 0.8 volumes of isopropanol. The DNA pellet was recovered by spooling on a glass rod, and rehydrated in 4 ml of buffer D (50 mM Tris, 1 mM EDTA, 200 μg/ml RNAse A, pH 8.0) and incubated overnight at 4°C. The solution was extracted once with an equal volume of chloroform, then precipitated with 0.8 volumes of isopropanol. The DNA pellet was recovered by centrifugation, washed once with 70% ethanol and stored dry at -20°C.

For growth curves, the strains (SS9, DSJ4, 3TCK) were grown at 9°C in polyethylene bulbs at different pressures (0.1, 15, 30, 45 and 75 MPa). At time intervals, one bulb was removed and its optical density at 600 nm was recorded. The growth rate was calculated from the log-portion of the curve obtained.

Detection of the 80 kb plasmid in the different *P. profundum *strains was done using primers

SS9PLAS1F (5'-ACAAGAGGCAGCAAAAAGACTAAC-3'),

SS9PLAS2R (5'-TGCCGCACAGGTAATGATAGGATG-3'),

SS9PLAS3F (TCAGTGCATCGCTAGGGTTAGACT-3'),

SS9PLAS4R (AAAGCATTATGAAAAATTGGTAGA).

The PCR cycle for amplification was 94°C for 5 min, followed by 30 cycles of 94°C for 30 s, 52°C for 30 s, 72°C for 1 min, and a final extension at 72°C for 7 min.

### DNA microarray preparation

Most of the microarray clones (2174) identifying a single ORF were selected from a small-insert genomic library, representing 1264 individual ORFs. In order to obtain a general overview of the SS9 genome we selected also 2227 non overlapping clones from a large-insert library, representing other 3488 ORFs. Clones were selected from 384 well plates using Biomek 2000 workstation and were inoculated in 96 well plates with 100 μl of LB/Ampicillin (50 μg/ml). Clones were grown overnight and then 1 μl was used for PCR amplification in 96 well plates. PCR amplification mix (one sample volume) (H_2_O mQ AF 47.616 μl; buffer 10 × 6 μl; MgCl_2 _25 mM 3.6 μl; dNTPs 10 mM 1.32 μl; primer -21M13 100 μM 1.32 μl; primer M13REV 100 μM 1.32 μl; Taq polimerase 0.2 μl; total volume 59 μl). The PCR cycle for amplification was 96°C for 5 min, followed by 35 cycles of 96°C for 25 s, 56°C for 30 s, 72°C for 2 min and a final extension step at 72°C for 10 min.

PCR products were purified using ethanol precipitation. PCR reactions were transferred to 96-well U-bottom tissue culture plates (Costar #3790) and 1/10 vol. 3 M sodium acetate (pH 5.2) and 2.5 volumes ethanol were added to the PCR. Plates were mixed by inversion and stored at -20°C overnight. Plates were centrifuged in Sorvall RC-3B at 3500 rpm for 1 h (RCF = 3565 g) and supernatant was poured off from plates. PCR products were washed with 100 μl of ice-cold 70% ethanol and centrifuged again for 30 min. Pellets were dried in speed-vac for 10 min. Before use, PCR products were resuspended in 15 μl 3 × SSC overnight using Titramax 101 plate shaker (Heidolph). For expression microarray experiments amplicons were arrayed onto MICROMAX™ Glass Slides (PerkinElmer Life Sciences, Inc.) using "MicroGrid II" spotter (Biorobotics, Genomic Solutions^®^) equipped with 16 pins. Microarrays have a spotted area of 18 mm × 18 mm and are composed by 16 subarrays (4 metacolumns and 4 metarows) containing three replicates of each spot. Spots have a diameter of approximately 80 μm and were spaced 150 μm. For genomic microarray experiments amplicons were arrayed onto MICROMAX™ Glass Slides (PerkinElmer Life Sciences, Inc.) using "GenPack Array21" spotter equipped with 32 pins. Microarrays have a spotted area of 36 mm × 18 mm, are composed by 32 subarrays (4 metacolumns and 8 metarows) containing two replicates of each spot. Spots have a diameter of approximately 130 μm and were spaced 250 μm.

### Nucleic acid labeling and hybridization conditions

#### Expression experiments

For microarray analysis at different pressure, bacterial cultures were grown as explained above at two different conditions (0.1 MPa/16°C and 45 MPa/16°C) and were queried against a common reference culture (28 MPa/16°C). For temperature microarray experiments bacterial cultures were grown at 0.1 MPa/4°C and were queried against a culture grown at 0.1 MPa/16°C. For each growth condition total RNA was extracted from three independent cultures using trizol (Gibco) and RNeasy columns (Qiagen). Genomic DNA was removed using DNAsi (Ambion). For microarray expression experiments 2 μl of random hexamer primers (3 mg/ml) were added to 20 μg of total RNA, volume was brought to 18.5 μl with RNase-free water and were labeled with Cy3 and Cy5 using an aminoallyl indirect labeling method according to the TIGR protocols with some minor modifications [48]. Solution was mixed well and incubated at 70°C for 10 min. After denaturation RNA was snap-freezed in dry ice/ethanol bath for 30 s, then it was centrifuged briefly at 10,000 rpm and maintained at room temperature. 6 μl of 5 × First Strand buffer, 3 μl of 0.1 M DTT, 0.6 μl of 10 mM dATP, dCTP and dGTP, 0.9 μl 10 mM dTTP, 0.6 μl 10 mM dUTP-AA, and 2 μl of SuperScript II RT (200U/ μl) (Invitrogen) were added to the solution that was mixed gently and incubated at 42°C for 3 h. To hydrolyze RNA 3 μl of NaOH 1 M and 0.6 μl of 500 mM EDTA were added to the mixture that was incubated at 65°C for 15 min in a water bath. 3 μl of 1 M HCl and 8.5 μl of 2 M HEPES (SIGMA) was added in order to neutralize pH. Removal of unincorporated aa-dUTP and free amines was made using Microcon YM-30 Cleanup method (Millipore). Sample volume was reduced to 9 μl in a speed vac and 1/10^th ^volume of carbonate buffer (Na_2_CO_3 _1 M, pH 9) was added to the cDNA and this solution was used to resuspend the Cy- NHS-ester dye. Reaction was incubated for 1 h in the dark at room temperature. Labelled cDNA was purified using the GenEluteTM PCR Clean-up kit (SIGMA). Analysis of labelled cDNA was made using a 50 μl quartz MicroCuvette (Beckman) to analyze the entire undiluted sample in a spectrophotometer. After analysis the two differentially labeled probes (Cy3 vs. Cy5) were combined and precipitated with NH_4_Ac and ethanol according to standard protocols. DNA was recovered by centrifugation, and the pellet was washed twice with 70% EtOH. The pellet was briefly air dried, then resuspended in hybridization buffer containing 50% formamide, 5 × SSC, 0.1% SDS, 100 ng/μl SS-DNA, 5 × Denhardt's solution. Labeled DNA was heated to 95°C for 2 min and chilled on ice before use in hybridization. Hybridization was performed over night in hybridization chamber (GeneMachines) at 42°C using coverslip. After hybridization microarrays were washed two times for 5 min with 1 × SSC, 0.1% SDS pre-heated at 42°C, two times for 5 min with 0.2 × SSC, 0.1% SDS at room temperature, two times for 5 min with 0.2 × SSC at room temperature and finally two times for 5 min with SSC0.1 × at room temperature.

#### Genomic experiments

For a 50 μl reaction, 10 μg of genomic DNA (gDNA) was combined with 4 μg of random hexamer primers and heated to 95°C for 5 min. After denaturation gDNA was snap-freezed in dry ice/ethanol bath for 30 s, was centrifuged briefly at 10,000 rpm and mantained at room temperature. 1.5 μl of 10 mM dATP, dCTP and dGTP, 0.9 μl 10 mM dTTP, 0.6 μl 10 mM dUTP-AA, 3 μl of Eco Pol buffer and 15 U of the Klenow fragment of *Escherichia coli *polymerase (NEB) were added to the reaction. The reaction was placed at 37°C for 2 h. Labeled DNA was then purified and labeled as described in the previous section. After precipitation DNA was recovered by centrifugation, and the pellet was washed with 70% EtOH and re-centrifuged.

Hybridization and post-hybridization washes were performed as described in the previous section "expression experiments".

### Image acquisition and analysis

Arrays were scanned with a ScanArray^® ^Lite scanner (PerkinElmer Life Sciences, Inc.). Hybridization signals were quantified using QuantArray software (PerkinElmer Life Sciences, Inc.). Data representing weak signals (median pixel intensity lower than 400 in both channels) were removed. Signal intensities were normalized using MIDAS V2.15 [49] and used to generate relative hybridization ratios (query/reference).

#### Expression experiments

normalization was performed using loc fit normalization (LOWESS) normalizing each single microarray block separately. The ratios from a maximum of nine data points (triplicate spots, hybridizations performed in triplicate) were analyzed with SAM software [50]. When less than six over nine data points existed, the clone was treated as data missing and was excluded from SAM analysis. Only clones having log_2 _ratio higher than 0.7 or lower than -0.7 and having d (the T-statistic value) higher than +2.3 or lower than -2.3 were considered. Differentially expressed clones spanning a single ORF were used for the identification of differentially expressed genes and the other clones were used in order to confirm or reject data obtained. All the fluorescence intensity data were used after normalization for the analysis of absolute gene expression on the two chromosomes.

#### Genomic experiments

analysis was performed as described for expression analysis but only clones having log_2 _ratio higher than 1 or lower than -1 and having d (the T-statistic value) higher than +2.7 or lower than -2.7 were considered.

### GC content, codon bias and genomic signature analysis of SS9 genome

GC content reported in Figure 2 is calculated as the G+C frequency considering 5 kb windows with 0.1 kb shift.

Codon bias and GC frequency in third position (S3%) in SS9 genes longer than 200 codons was calculated using a home-made perl script based on formula described in [9].

Genomic signature was calculated as described by Karlin S. [7] considering tetranucleotide frequency calculated as the odds ratio ρ_XYZK _= *f*_XYZK_/*f*_X_*f*_Y_*f*_Z_*f*_K _where *f*_XYZK _is the frequency of the tetranucleotide XYZK in the sequence under study. For double-stranded DNA sequences, symmetrized version ρ*XYZK is computed from frequencies of the sequence concatenated with its inverted complementary sequence. ρ*_XYZK _was calculated for the whole genome (c*_XYZK_) and for 5 kb windows (r*_XYZK_) (with a 0.5 kb step). The delta distance, calculated as δ* = 1/256 Σ |r*_XYZK_-c*_XYZK_|, was plotted in Figure 2 (10^th ^circle).

## Authors' contributions

SC conceived of the study, performed the microarray experiments and analysis, and drafted the manuscript. AV participated in the design and coordination of the study, revised the manuscript and participated in the interpretation of the microarray results. NV participated in the design of the study, performed the bioinformatic analysis (GC content, codon usage, genomic signature, phage identification and UCSC genome browser implementation). FML participated in the design of the study, carried out the *P. profundum *coltures, growth curves, phylogenetic studies, RNA and gDNA extraction and revised the manuscript. MD and FS participated in microarray production and in the interpretation of the microarray results. AC participated in Gene Ontology annotation. GM participated in microarray experiments. GB participated in the interpretation of the microarray results. GV and DHB participated in the design and in the coordination of the study, in the interpretation of the microarray results and revised the manuscript. All authors read and approved the final manuscript.

## Supplementary Material

Additional File 1 Codon bias relative to average gene versus third position GC content in eight variable regions of the *Photobacterium profundum *SS9 genome. In these graphs are represented only ORFs longer than 200 codons. Eight variable regions of the SS9 genome are considered, one for each graph. In red are highlighted ORFs located in regions that were found absent in 3TCK/DSJ4 genomes using comparative genomic hybridization experiments. A large number of ORFs are positioned in left and right horn of the graphs, a strong indication that they belong to laterally transferred regions. Click here for file

Additional File 2 Detection of the 80 kbp plasmid in two different *P. profundum *strains. Comparison of strain TW30 (lanes A, B) with parental strain DB110 (lanes C, D) with plasmid specific primers. Lane E: no DNA control for PCR. Lane F: 2-log ladder marker (New England Biolabs). Click here for file

Additional File 3 List of differentially expressed genes obtained in microarray experiments. This table reports only the ORFs identified univocally by the microarray clones and only those having log_2 _ratio ≥|0.7| and "d score" obtained from SAM analysis = |2.3| (indicated with "1" in columns 7–12). Table columns report respectively: (1, 2, 3) ORFs differentially expressed in the three conditions under analysis, (4, 5) clones that were found absent in 3TCK and DSJ4 strains, (6) ORFs that were found absent in 3TCK strain ("3TCK"), in DSJ4 strain ("DSJ4") or in both strains ("3TCK-DSJ4"), (7) ORFs up-regulated at 28 MPa, (8) ORFs down-regulated at 28 MPa, (9) ORFs up-regulated at 4°C, (10) ORFs down-regulated at 4°C, (11) ORFs up-regulated at 45 MPa, (12) ORFs down-regulated at 45 MPa, (13) ORF annotation, (14) spotted microarray clones identifying each ORF, (15, 16), (17, 18), (19, 20) ("log_2 _ratio" and "d value") mean value of expression differences calculated as log_2 _[(Σ fluorescence value at 28 MPa)/(Σ fluorescence value at 0.1 MPa)] and the "d" statistic value obtained from SAM analysis. Each ORF is identified by one or more clones and not all ORFs identified from each clone are reported in the table (for a complete picture see the UCSC genome browser [12]). Row data obtained from microarray experiments were submitted to ArrayExpress database at EBI with accession numbers E-MEXP-210, E-MEXP-348, E-MEXP-374, E-MEXP-375, E-MEXP-376. Click here for file

Additional File 4 List of ORFs absent only in 3TCK strain. Data obtained from genomic comparison experiments between 3TCK (the pressure sensitive strain) and SS9 strain. In this table are reported ORFs that were found absent only in 3TCK and not in DSJ4 strain. In order to obtain a more reliable result, clones obtained from SAM analysis of 3TCK strain were filtered considering only those having log_2 _ratio higher than +1 and d statistic value higher than +1.8. Using self-written PERL scripts, from the "3TCK clones" set, were subtracted the clones that were found absent also in DSJ4. In DSJ4 strain we used less stringent criteria and we considered all clones having log_2 _ratio higher than 0.5. Clones absent both in 3TCK and DSJ4 were discarded. Clones absent only in 3TCK were associated with ORFs position and the list of ORFs obtained were manually verified. Table columns show: (1) the locus name, (2) the ORF description, (3) the TrEMBL code, (4) the microarray clone/s overlapped to the ORFs, (5) the log_2 _ratio obtain from comparison with SS9 genome (reference), (6) the "d" statistic value obtained from SAM analysis and (7) the result obtained in microarray expression experiments. In the first column large groups of ORFs that are adjacent in the SS9 genome are highlighted using bold character. Click here for file

Additional File 5 List of Gene Ontology categories of differentially expressed genes. In this table differentially expressed genes reported in Additional file 3 are categorized in Gene Ontology classes using GoMiner software. Only highly significant GO classes are reported. Table columns show: (1) the Gene Ontology ID, (2) the total number of *P. profundum *SS9 genes belonging to each class, (3, 4, 5) the "p statistic value" of down-regulated, up-regulated and differentially expressed genes, (6) the Gene Ontology term, (7) the "UniProtKB/TrEMBL" primary accession number, (8) the ordered locus name, (9) the name of differentially expressed genes belonging to each Gene Ontology category and (10) their "up-" or "down-regulation". Click here for file
