# Supplementary figures and images for: Laterally transferred elements and high pressure adaptation in Photobacterium profundum strains
Source: BMC Genomics. 2005 Sep 14;6:122. doi: 10.1186/1471-2164-6-122 (PMC1239915; doi:10.1186/1471-2164-6-122)

## Additional file 1

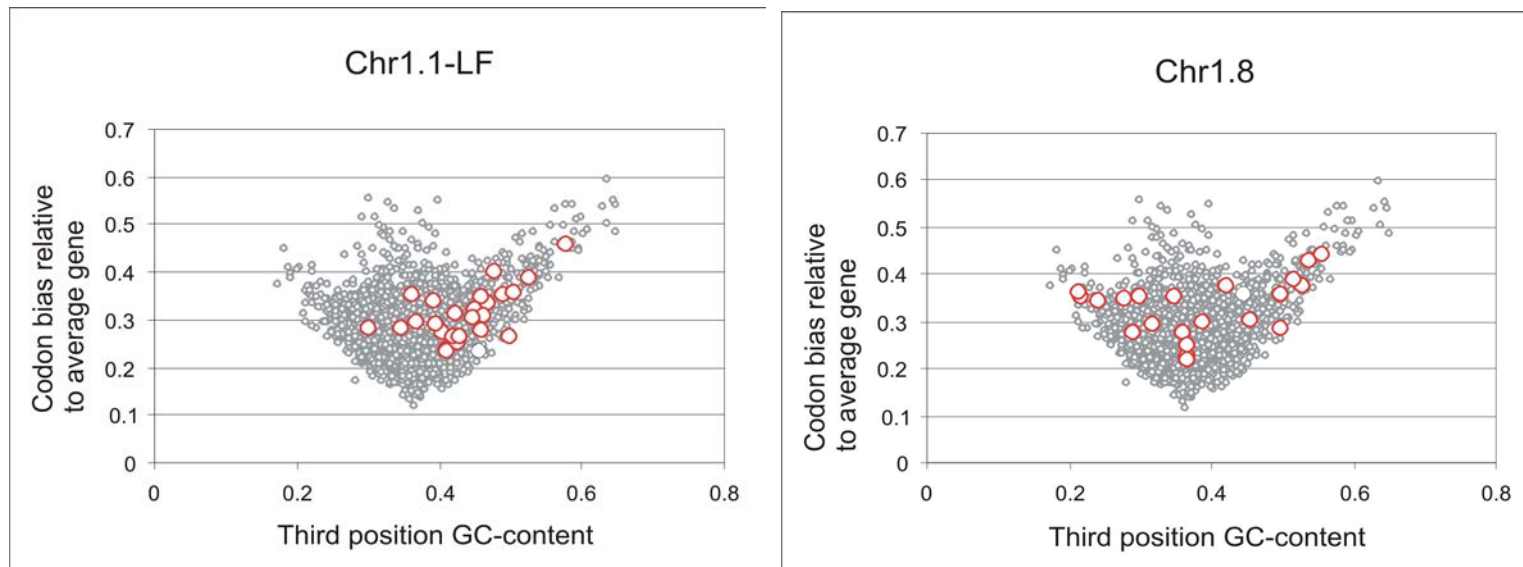

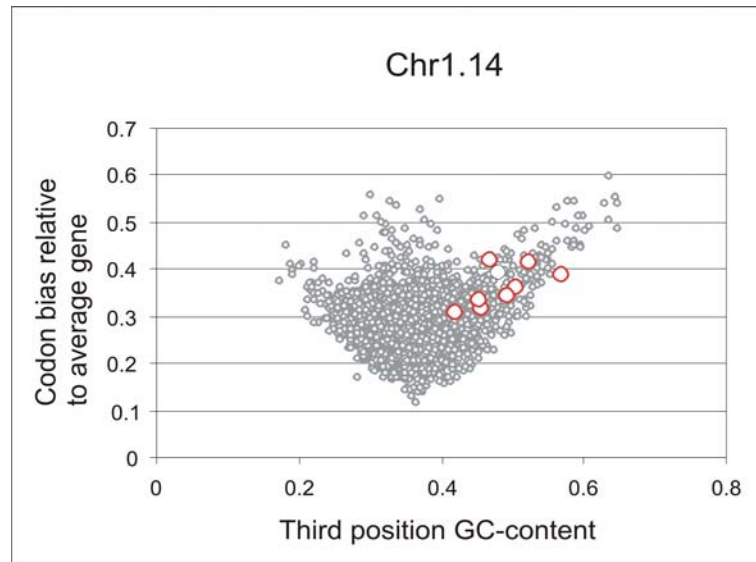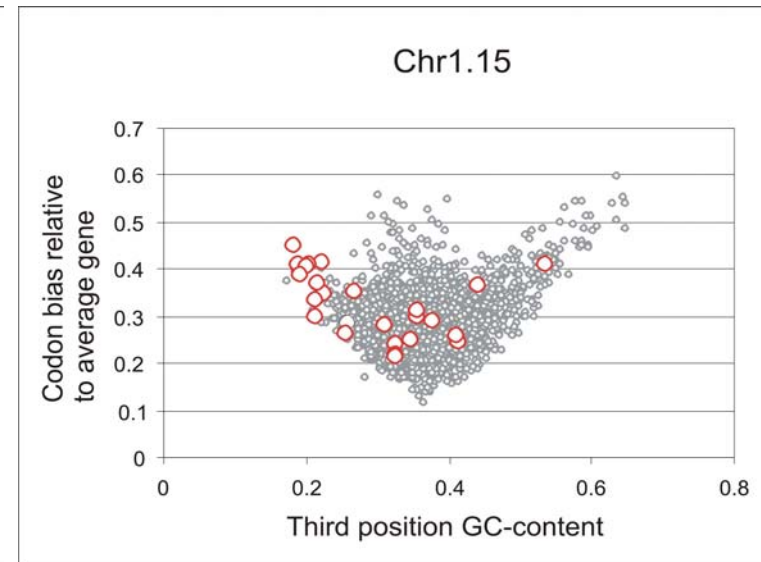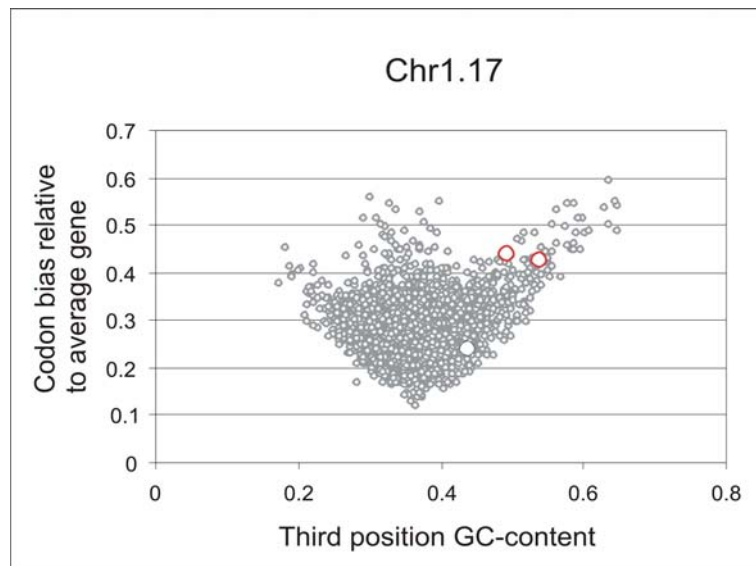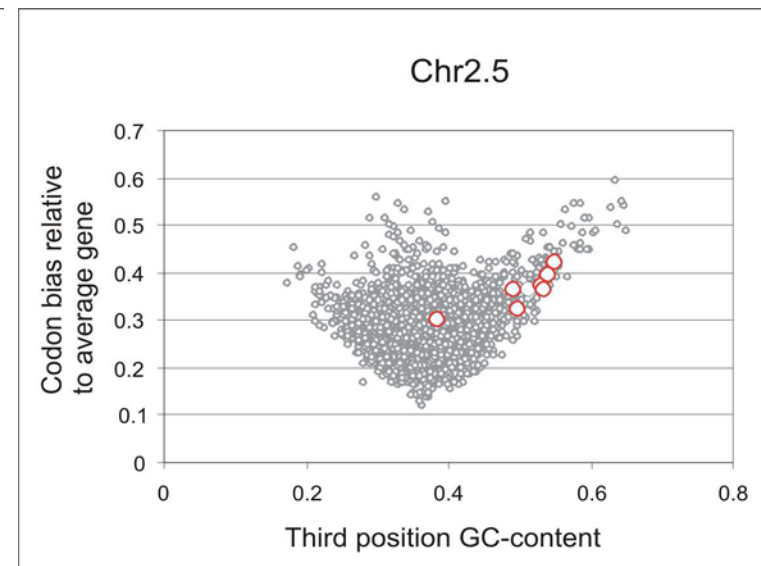

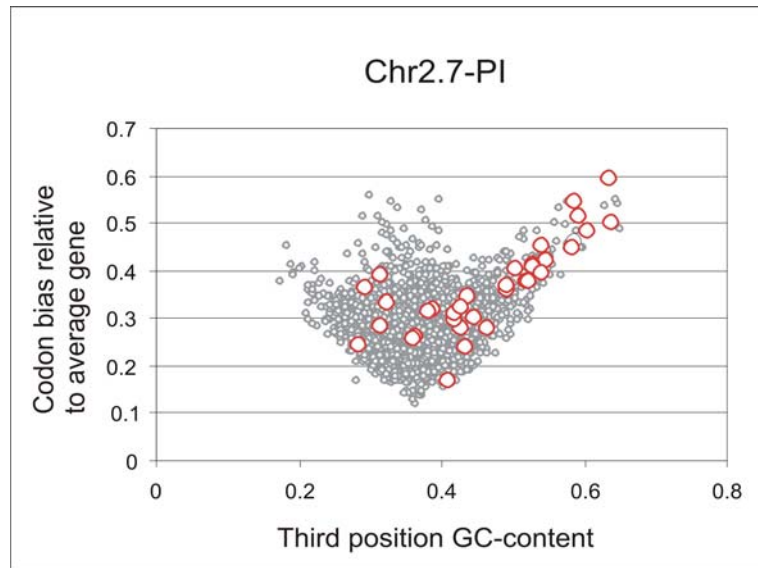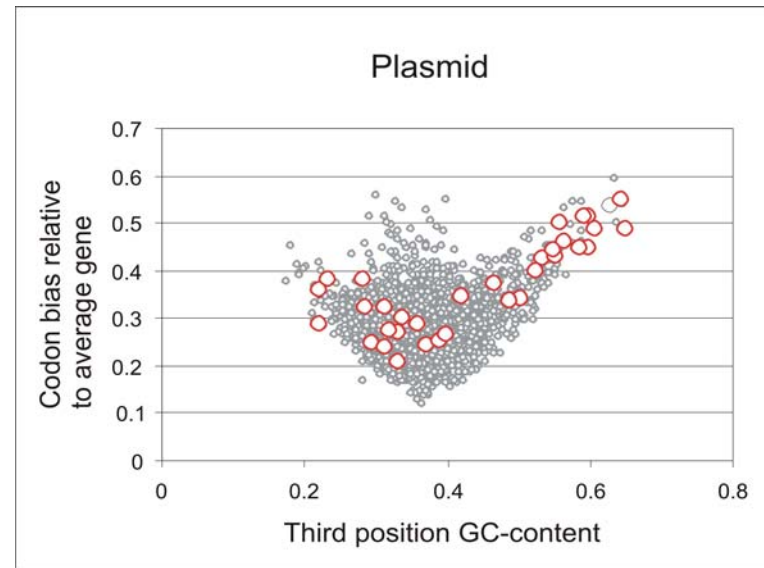

Supplement: Additional File 1 — Codon bias relative to average gene versus third position GC content in eight variable regions of the Photobacterium profundum SS9 genome. In these graphs are represented only ORFs longer than 200 codons. Eight variable regions of the SS9 genome are considered, one for each graph. In red are highlighted ORFs located in regions that were found absent in 3TCK/DSJ4 genomes using comparative genomic hybridization experiments. A large number of ORFs are positioned in left and right horn of the graphs, a strong indication that they belong to laterally transferred regions. [file 1471-2164-6-122-S1.pdf]

## Additional file 2

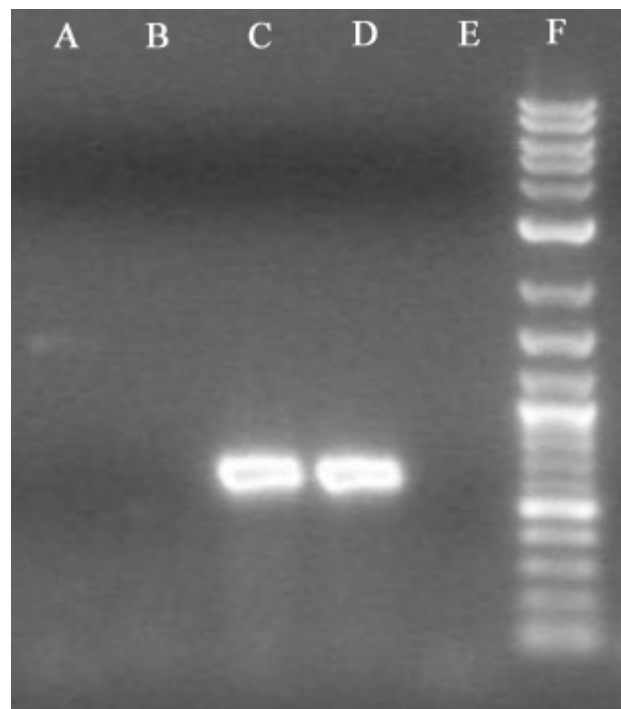

Supplement: Additional File 2 — Detection of the 80 kbp plasmid in two different P. profundum strains. Comparison of strain TW30 (lanes A, B) with parental strain DB110 (lanes C, D) with plasmid specific primers. Lane E: no DNA control for PCR. Lane F: 2-log ladder marker (New England Biolabs). [file 1471-2164-6-122-S2.pdf]
